# Emerging roles of activating transcription factor 2 in the development of breast cancer: a comprehensive review

**DOI:** 10.1093/pcmedi/pbad028

**Published:** 2023-10-25

**Authors:** Ahmed Amarah, Ahmed Adel Elsabagh, Amr Ouda, Omar Karen, Khaled Ferih, Ibrahim Elmakaty, Mohammed Imad Malki

**Affiliations:** College of Medicine, QU Health, Qatar University, P.O. Box 2713, Doha, Qatar; College of Medicine, QU Health, Qatar University, P.O. Box 2713, Doha, Qatar; College of Medicine, QU Health, Qatar University, P.O. Box 2713, Doha, Qatar; College of Medicine, QU Health, Qatar University, P.O. Box 2713, Doha, Qatar; College of Medicine, QU Health, Qatar University, P.O. Box 2713, Doha, Qatar; College of Medicine, QU Health, Qatar University, P.O. Box 2713, Doha, Qatar; College of Medicine, QU Health, Qatar University, P.O. Box 2713, Doha, Qatar

**Keywords:** ATF2, breast cancer, oncogene

## Abstract

Activating transcription factor 2 (ATF2) is a member of the leucine zipper family of DNA binding proteins that are responsible for regulating various genes that play an essential role in major biological and cellular functions. Since ATF2 plays a vital role in cellular proliferation and apoptosis, it is believed that it greatly affects the development of breast cancers. However, its exact role in breast cancer is incompletely understood. It remains a subject of debate, ambiguity, and continuous research. Several studies have suggested the role of ATF2 as an oncogene, promoting cellular proliferation and worsening the outcome of cancers. In contrast, other studies have postulated that ATF2 plays a tumor suppressive role in estrogen receptor-positive breast cancer. The ambiguity surrounding its role in breast cancer is the reason why there is an influx of recent studies and research in this area. In this narrative review, we investigate several studies that have been published about the role of ATF2 in breast cancer. We also explore studies that have examined the association between ATF2 and endocrine therapy resistance. ATF2 has been suggested to modulate estrogen receptor (ER) expression and activity, potentially affecting tamoxifen sensitivity in breast cancer cells. Therefore, the role of ATF2 in DNA repair mechanisms and drug resistance has been deeply explored in this review. Additionally, there are numerous ongoing clinical trials exploring the effect of targeting ATF2 pathways and mechanisms on the outcome of breast cancers, some of which we have discussed. The studies and clinical trials that are being conducted to understand the multifaceted role of ATF2 and its signaling pathways may provide valuable insight for developing efficient targeted therapeutic solutions to enhance the outcomes of breast cancer and overcome endocrine resistance. We suggest further research to elucidate the dual roles of ATF2 in breast cancer and potential therapeutic therapies for its treatment.

## Introduction

Breast cancer is the most diagnosed cancer in women in most countries. It is the second most common cause of death in women from cancer in the world.^1^ It is an aggressive disease that usually evolves silently. It accounts for 11.7% of cancer cases diagnosed worldwide.^2^ It also accounts for ∼6.6% of cancer deaths worldwide. It may be caused by both hereditary mutations, which account for ∼5% to 10%, but nonhereditary causes have also been identified in several studies.^3^ Its incidence has also shown a rise in consecutive generations.^4^ Due to its silent nature, it is usually discovered during screening or following an accidental finding. Earlier diagnosis usually indicates better prognosis.^5^ There is an increase in prevalence in countries witnessing socioeconomic growth, which can be attributed to the rise of several risk factors, including nulliparity and delay in childbearing age, oral contraceptive use, hormone replacement therapy, and increase in average weight and fat distribution. The decrease in postmenopausal hormonal exposure may have led to a reduction in breast cancer cases in developed countries.^2^ The occurrence of breast cancer in men is rare (∼1% of cancers in men and 1% of breast cancer cases are men).^6^ In this review, we will discuss endocrine treatment of breast cancers and the role of activating transcription factor 2 (ATF2) in cancer and its treatment.

Typically, endocrine therapy in breast cancer is indicated for hormone receptor-positive cancer. This aids in reducing recurrence specifically in node-positive disease.^7,8^ Using tamoxifen as a treatment lowers the mortality rate of breast cancer with an absolute risk reduction of 9.2% in 15 years and an number needed to treat (NNT) of 11.^7,9^Five years of adjuvant tamoxifen has demonstrated a reduced risk of cancer recurrence by ∼50% in 0 to 4 years.^9,10^ Combined induction of both endocrine therapy and chemotherapy may help reach optimum results.^9,10^ Patients who are <35 years old or receive chemotherapy in an adjuvant setting receive the same endocrine therapy regimen plus added ovarian suppression, which could be either chemical or surgical.^11^

Historically, cancer genes were classed as tumor suppressors or oncogenes. Nevertheless, it has been evident from research in the last decade that genes may behave in both ways according to the cell type and stimulus.^12^ One of these genes is ATF2, a member of activator protein 1 (AP-1).^13^ It is found on chromosome 2q32. When translated, it gives a large protein with 505 amino acids.^14^ It stimulates transcription by binding to cyclic AMP-response elements (CREs) and then forming a homodimer or heterodimer with c-Jun.^15^ Other domains include the N-terminal transactivation domain, the zinc finger, basic leucine-zipper (bZIP) domain, and nuclear localization and export signals. Roles of ATF2 also include working as an epigenetic regulator through acetylation of histones H2B and H4 via a histone acetyltransferase domain. The role of ATF2 in suppressing or promoting tumor aggressiveness depends on subcellular localization.^16^ It also plays a role in the transformation of epithelial cells into highly migrating mesenchymal cells in cases of cellular stress, which promotes tumor aggressiveness.^17,18^ Its role is not only recognized and studied in breast cancer. For instance, it has been shown that ATF2 knockout might lead to tumor suppression or promotion depending on the model of mice used in the experiment.^19^ Its roles also include the regulation of several genes responsible for cell multiplication, apoptosis, transformation, and inflammation.^16^ These genes include MMP-2 and MMP-9 in MCF10A breast epithelial cells, in which ATF2 induces their migration and promotes their invasiveness.

### Regulation of ATF2 expression and activity by phosphorylation

**Figure 1.**
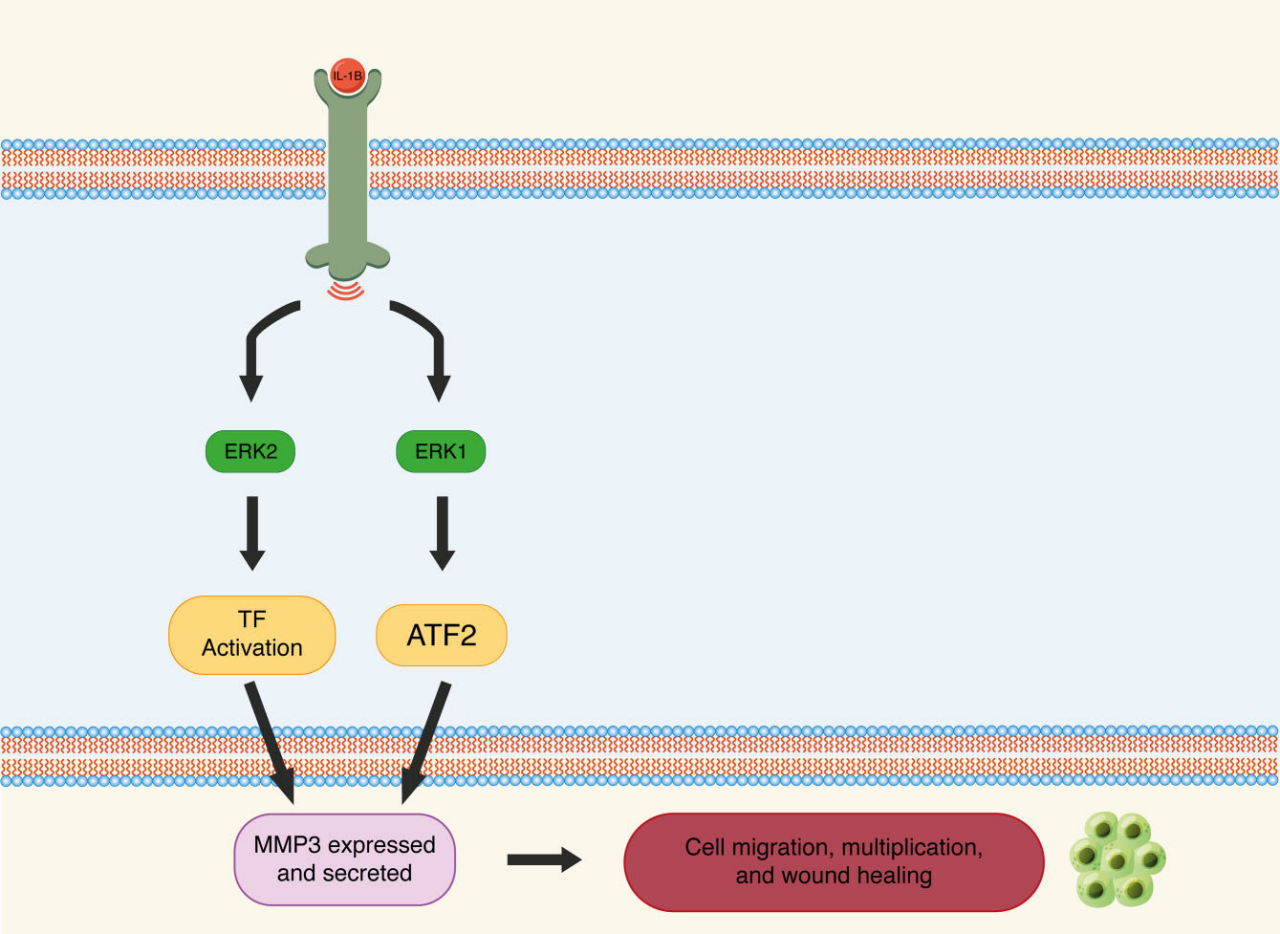
An illustration showing the ERK1/ATF2 pathway that upregulates MMP3 leading to several cellular effects. TF, transcription factors.

**Figure 2.**
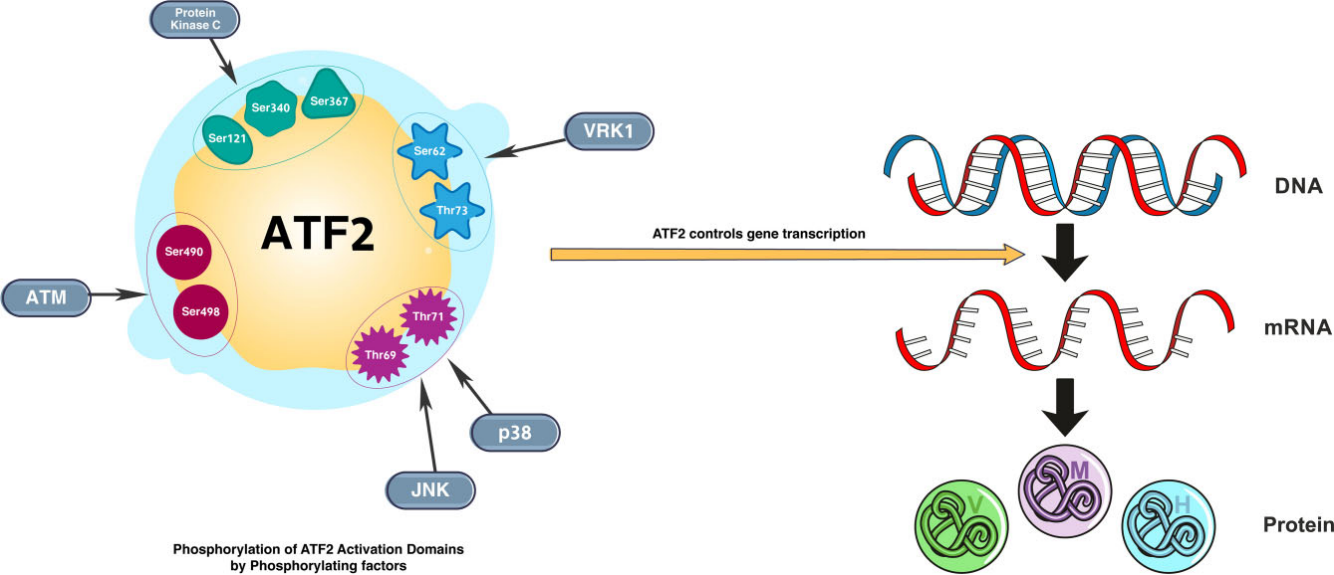
An illustration depicting the phosphorylation of ATF2 activation domains by phosphorylating factors and the consequent actions of ATF2 on gene transcription. p38, p38 MAPK.

ATF2 belongs to the ATF and cAMP response element binding protein (CREB) group of bZIP transcription factors. It is also a member of the leucine zipper family of DNA binding proteins.^20^ These proteins are responsible for regulating numerous genes that play vital roles in cell growth, proliferation, cell death, inflammation, and DNA damage repair.^21^ ATF2 controls gene transcription as a homodimer, but it also operates as a heterodimer with other ATF and AP-1 family members.^22^ The attachment of these homo- and hetero-dimers to ATF/CRE causes changes that influence chromatin remodelling, transcription, and the DNA damage response.^20^ The attachment is dependent on the binding partner of ATF2.^16^ It can bind to CRE consensus sequences (5-TGACGTCA-3) or to AP-1 consensus sequences (5-TGACTCA-3).^22^ For the transcriptional properties of AFT2 to be activated, some phosphorylation events need to occur and are usually in response to extracellular stresses such as hypoxia, reactive oxygen species, inflammatory cytokines, ultraviolet, and increased osmolarity.^23^ The activation domain needs to be phosphorylated, particularly the two threonine residues (Thr69 and Thr71).^24^ There are several phosphorylating factors that target specific ATF2 phosphorylation sites.^20^ For instance, p38 mitogen-activated protein kinase (MAPK) and c-JUN N-terminal kinase (JNK) are responsible for phosphorylating both Thr69 and Thr71. However, Epidermal growth factor phosphorylates only Thr71 via extracellular signal-regulated kinase (ERK)1/2 (Fig. 1).^25^ Several articles have also reported that additional signaling pathways can play a role in the phosphorylation of AFT2, such as its activation by human vaccinia-related kinase 1 (VRK1) at two sites, Ser62 and Thr73, in its activation domain, as shown in Fig. 2.^16,26^ Protein kinase C also plays a role as it phosphorylates sites such as Ser121, Ser340, and Ser367.^20^ In addition, AFT2 is also activated by growth factors via guanine nucleotide dissociation stimulator RalGDS, the Ras/ERK pathway, and Src.^27^ Finally, Ser490 and Ser498 are phosphorylated by ataxia telangiectasia mutated (ATM) protein kinase.^20^ It is believed that this is vital for intra-S-phase checkpoint activation following ionizing radiation. ATM protein kinase directs its localization into DNA repair foci, where it colocalizes with DNA repair machinery components such as Rad50, Nbs1, and Mre11.^22^ The recruitment of ATF2 to irradiation-induced foci, irrespective of its transcription-activating capabilities, suggests that it may play a role as a sensor/adaptor early in the DNA damage response.^28^ Compared to wild-type ATF2 mice, genetically engineered mice with mutant ATM phosphoacceptor sites demonstrated enhanced susceptibility to ionizing radiation. Furthermore, these ATF2 mutant animals had an increased frequency of spontaneous and chemically driven tumor formation, indicating the relevance of ATF2 phosphorylation by ATM in the acute cellular response to DNA damage and genomic stability maintenance.

### ATF2 in human cancers

In this review, we will focus on the role of ATF2 in the development of breast cancer. However, ATF2 is also involved in the tumorigenesis of several other cancers, including skin cancers, leukemia, renal cell carcinoma, brain tumors, and cervical cancer (Table 1). As mentioned previously, ATF2 may also act as a tumor suppressor when activated. ATF2 plays a major role in the breakdown of the extracellular matrix by regulating matrix metalloproteases (MMPs). MMP-3 is specifically found in dermal fibroblasts and is induced by interleukin 1β (IL-1β). This process is performed by activation of the ERK1/ATF2 axis. Hence, ATF2 may be implicated in the development of disease and conditions related to MMP-3 overexpression, such as skin aging, arthritis, vascular diseases, and neurodegenerative diseases.^29^ In addition, it is implicated in several cancers, such as breast, colorectal, lung, and pancreatic cancer. It was also found in recent studies, including a 2022 study by Deng *et al.*, that MMP-3 blockade inhibited cancer progression in cellular and animal models with pancreatic ductal carcinoma. It also reduced the rates of resistance to therapy by gemcitabine.^30^ In addition, Gonzalez-Avila *et al*. studied the effect of MMPs in lung adenocarcinoma and showed a positive correlation between MMP-3 overexpression and cancer aggressiveness. They also concluded that they might be a potential target for future therapeutic strategies.^31,32^ Wen *et al*. also found that MMP-3 may also be implicated in colorectal cancer invasion and metastasis. They found that HDAC11, which downregulates MMP-3, was underexpressed in patients with colorectal cancer.^33^

**Table 1.** Role of ATF2 in tumorigenesis of human cancers.

Cancer	Pathway	Effect
Breast, colorectal, lung adenocarcinoma; pancreatic ductal carcinoma	MMP-3 dysregulation and overexpression	MMP-3 has direct effects on cancer progression and increasing resistance to chemotherapy. This promotes more aggressive forms of cancer and increases probability of metastasis.
T-ALL	MAP2K7 pathway	ATF2 acts as a downstream agent to MAP2K7 alongside c-jun, which increases susceptibility to leukemia.
RCC (with increased chemoresistance)	ATF2 overexpression in renal cells	Leads to cell survival and chemoresistance.
Invasive melanoma	ATF2 is activated by PKCε.	Directly increases melanoma cell's migratory and invasive behaviors by decreasing cell fucosylation.
Melanoma	ATF2 reduces BRAF inhibitors.	Induces apoptosis in melanoma cells and reduces resistance rates.
Liver tumors	ATF2 in a downstream target for JNK, regulation of cell growth, and apoptosis	Leads to suppression of liver tumorigenesis.

T-ALL, T-Cell acute lymphoblastic leukemia; RCC, renal cell carcinoma; PKCε, protein kinase Cε.

**Figure 3.**
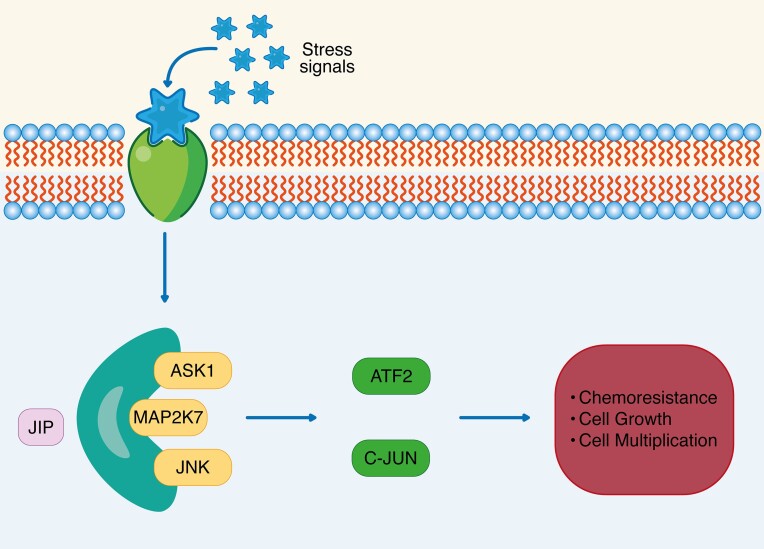
An illustration showing how the JIP complex is activated by stress signalling molecules (including ASK1, MAP2k7, and JNK), inducing the activation of ATF2 and C-JUN, which was shown to increase cellular mechanisms like chemoresistance, cell growth, and multiplication, which may enhance tumor progression. JIP, JNK-interacting protein; MAP2k7, mitogen-activated protein kinase; ASK1, apoptosis signal-regulating kinase 1.

Moreover, ATF2 was shown to induce cancer genesis and progression in T-cell acute lymphoblastic leukemia (T-ALL). This is because ATF2 is one of the downstream effectors in the MAP2K7 pathway, as shown in Fig. 3, which was implicated in the promotion of T-ALL. It was also shown that in mouse models, inhibition of this cascade may help limit the progression of leukemia.^34^ Another study has shown the overexpression of ATF2 in renal cell carcinoma. ATF2 leads to cell survival and chemoresistance in cancerous cells. The authors of the study were able to make cells more chemosensitive after targeting miR-451 to reduce ATF2. They concluded that this method might be beneficial to enhance chemosensitivity, leading to better chemotherapy outcomes in patients with renal cell carcinoma.^35^ ATF2 has also been studied in melanoma. Lau *et al*. have found evidence that demonstrates the control of migratory and invasive behaviors of melanoma by ATF2. They found that ATF2 activation via protein kinase Cε (PKCε) phosphorylation may have led to more advanced and metastatic forms of melanoma. Increased PKCε and ATF2 expression was correlated with increased cell adhesion and cell motility and decreased cell protein fucosylation. The restoration of fucosylation in mouse models also showed a reduction in distant metastasis.^36^

ATF2 may also be protective. Novel therapies are being developed in which ATF2 is used to induce apoptosis and reduce BRAF inhibitor resistance rates, leading to an increase in apoptosis of melanoma cells.^37^ In addition, Gozdecka *et al*. identified ATF2 as a downstream target of JNK, a protein kinase with roles in the regulation of cell growth, differentiation, and apoptosis. They found that this pathway may play an important role in the suppression of tumor formation in the liver and may provide a potential strategy to treat liver cancer.^38^

### Role of ATF2 in breast cancer

Despite all the studies, the definite role of ATF2 in breast cancer is still ambiguous, but research suggests that it may act as an oncogene.^22^ We will delve deeper into some studies done on the topic to give us a clearer picture of the evidence at hand. A study performed by Giannoudis *et al*. showed that ATF2 increases the transcription of genes such as MMP13, cyclin A, aromatase, and MMP2, which can contribute to breast cancer metastasis and proliferation. The increased transcription of MMP13 by AFT2 may play a role in facilitating the metastasis of breast cancer to bone. For cyclin A, it has been found that its transcription is led primarily by cJun-ATF2 dimers increasing cell proliferation.^21^ Evidence of increased aromatase transcription by ATF2 was shown in a study that concluded that there were increased quantities of phosphorylated ATF2 (pATF2) at the promoter of the aromatase gene responsible for estrogen synthesis after co-culturing with malignant epithelial cells in primary human adipose fibroblasts obtained from breast cancer patients.^39^ ATF2 is also thought to play a major role in breast cancer metastasis, as its phosphorylation (pATF2) has been demonstrated to enhance the transcription of MMP2, which boosts migration in HRas-transformed MCF10A human breast epithelial cells.^40^ ATF2 also combines with c Jun and c-Fos to facilitate HER2 activation of cyclooxygenase-2 (COX2), which is implicated in the growth and metastasis of cancer.^41^ Numerous research studies have indicated that v-src triggers the binding of ATF2 and CREB to the CRE/ATF site of the cyclin D1 gene, which results in the transcription of cyclin D1 in human breast cancer cells (MCF7).^21^ Furthermore, ATF2 potentially participates in pp60v-src signaling, a proto-oncogene that was found to accelerate the G1 phase progression of the cell cycle by inducing cyclin D1 protein levels in NIH3T3 cells.^42^ These collective findings strongly suggest that ATF2 plays a crucial role as an oncogene in breast cancer.

Another study investigated the effect of noxin on the ATF2 signaling pathway and its effect on breast cancer development.^43^ Noxin, which is also known as chromosome 11 open reading frame 82 or DNA damage-induced apoptosis suppressor, is linked to preventing cell death and promoting cell growth when the body is under stress. However, the exact role of noxin in regulating cell growth is still a subject of debate, and there have been no reports on its function and association with breast cancer. In this research, it was discovered that high noxin expression was linked to advanced tumor, lymph node metastasis, and poor overall survival of patients. Noxin was found to have increased levels of expression in breast cancer cells compared to normal breast cells. Noxin overexpression boosted tumor proliferation and maximized colony formation in MCF7 cells, while its depletion resulted in the opposite outcomes in MDA-MB-468 cells. Similarly, proliferation experiments showed that noxin boosted the growth of normal breast cells, and further testing revealed that cyclin D1 and cyclin E1 in MCF7 cells were upregulated when noxin was overexpressed but downregulated in MDA-MB-468 cells when noxin was depleted. Moreover, overexpression of noxin also increased the levels of phosphorylated p38 and ATF2 and lowered them when noxin levels were reduced. Inhibiting p38 countered the effects of noxin overexpression on cyclin D1, cyclin E1, and cell growth. Overall, the research indicated that noxin's role in worsening the outcomes of breast cancer was mainly through activating the p38-ATF2 pathway. As was evident in this study, inhibiting the p38-ATF2 pathway reversed the unfavorable outcomes of noxin on breast cancer cells, which may provide great insight into the development of new drugs that alter the outcomes of breast cancer. In contrast, another study indicated that noxin acts as a negative regulator of the p38-ATF2 pathway.^44^ It also indicates that the p38-ATF2 pathway may act as an apoptotic mechanism in breast cancer. Silencing of NOX caused apoptosis of non-small cell lung carcinoma A549 cells by activating the p38-ATF2 pathway. These contradictory roles of ATF2 may indicate that it has dual roles depending on the type of tumor and other factors affecting the tumor. Therefore, extensive research still needs to be done in this area.

A study by Tsai *et al*. experimented with the effect of timosaponin AIII on triple negative breast cancer cells (TNBC). It also experimented with the mechanism by which it can bring about its effect. The work found that TAIII caused a dose-dependent inhibition of hepatocyte growth factor-induced invasion activity in TNBC. This inhibition is believed to be accomplished through the activation of the ERK pathway, which in turn suppresses the nuclear expression of the ATF2 and COX genes. This has also been linked to the reduction in the activity levels of transcription factors such as AP-1, C/EBP, and CREB.^45^ Finally, Hayakawa *et al*. investigated the role of ATF2 in determining the chemoresistance of breast cancer cells to DNA-damaging agents such as cisplatin, actinomycin D, methionine-S-methyl sulfonium chloride, and etoposide.^46^ The study suggests that JNK leads to a drug resistance phenotype by activating ATF2, which plays an important role in mediating augmented DNA repair through a p53-independent mechanism. The article implies that the four DNA-damaging agents activate JNK, p38, and extracellular signal-regulated kinase (ERK), thereby increasing phosphorylation and ATF2-dependent transcriptional activity in human breast cancer BT474 cells. ATF2 can regulate numerous component genes that contain functional AP-1 and/or ATF/CREB binding sequences. This multicomponent system likely plays an important role in the complex cellular response of DNA damage repair.^46^ The results of this study indicate that ATF2 can cause resistance to DNA-damaging agents by stimulating DNA repair and mediating a chemotherapeutic drug resistance mechanism. This study also provides great insight into the role of ATF2 in endocrine treatment and drug resistance in human breast cancer cells, which may provide an important basis for other research to develop effective therapeutic solutions to treat drug-resistant cancers.

As evident from the studies performed, the exact role of ATF2 in the development of cancer is yet to be fully understood. There are contradictions regarding the exact role of ATF2, as some research suggests that it may have a dual role depending on the type of cancer and other affecting factors. Therefore, many clinical trials are being performed to further investigate this topic. Research in this area might provide great insight into potential therapeutic drugs that can target those pathways and help in the treatment of breast cancer. We performed a thorough search on clinicaltrials.gov, which yielded numerous clinical trials in progress/completed to test the impact of certain drugs on ATF2 pathways in the treatment of different types of cancers. Two clinical trials that focus on breast cancer specifically are discussed below.

One such study is from clinicaltrials.gov.^47^ As explained above, ATF2 is a transcription factor that plays major roles in regulating cell growth, differentiation, and apoptosis. Dysregulation of the MAPK-ATF2 signaling pathway has been linked with various diseases, including breast cancer. This clinical trial aims to investigate the efficiency of a combination therapy of fulvestrant and selumetinib in treating patients with advanced breast cancer that progressed after aromatase inhibitor therapy. Fulvestrant mainly blocks estrogen receptors, while selumetinib inhibits the MAPK pathway, which can result in the activation of ATF2. The study investigates whether the addition of selumetinib to fulvestrant can enhance progression-free survival compared to fulvestrant alone by targeting the MAPK-ATF2 signaling pathway. The primary endpoint of the study was progression-free survival at 6 months. Secondary endpoints include overall survival, objective response rate, and safety of the drug. The study enrolled ∼144 participants and was completed in 2022. The results showed that the combination of selumetinib and fulvestrant had worse efficacy parameters and progression-free survival than fulvestrant with placebo. The overall survival of patients did not show a significant difference between the study groups. The combination drug was also not tolerable for many patients, which caused an early reduction in the dose of the treatment or discontinuity of the treatment for some patients. One suggested hypothesis explaining the negative results of the combination therapy is that the inhibition of the MAPK pathway causes a shift and an increase in the other cellular pathways, such as the phosphatidylinositol 3-kinase pathway.^48^ This pathway is one of the most activated pathways in breast cancer and is believed to play a major role in endocrine resistance.^48^

Another study is “TAS-116 plus palbociclib in breast and Rb-null cancer”, which is a preclinical phase 1 study investigating the combination therapy of heat shock protein 90 (Hsp90) inhibitor TAS-116 and cyclin-dependent kinase 4/6 inhibitor palbociclib in the treatment of breast cancer and retinoblastoma-null cancer (Clinical trial—NCT05655598). TAS-116 is an inhibitor of the Hsp90 chaperone protein. This protein is involved in the activation of numerous oncogenic proteins, such as ATF2. The inhibition of Hsp90 is emerging as a promising therapeutic solution for cancer treatment; thus, there is a focus on Hsp90 inhibitors in several current clinical trials. Palbociclib is a selective inhibitor of CDK4/6 (kinases that play vital roles in the regulation of the cell cycle). It has proven efficiency in the treatment of hormone receptor-positive breast cancer. Therefore, it is approved for use in combination hormone therapy in this clinical trial. Investigating the possible synergy between TAS-116 and palbociclib in preventing the growth and survival of breast cancer and Rb-null cancer cells and enhancing their outcomes is the main aim of this study. The results of this study and similar studies may provide substantial insight into potential therapeutic strategies by targeting the Hsp90-ATF2 and CDK4/6 pathways in breast cancers.

The recent research findings presented in this review provide valuable insights into the evolving landscape of ATF2, particularly in the context of hormone-receptor-positive breast cancer and the intricate mechanisms involved in resistance and response to therapy. These studies collectively underscore the significance of novel substances targeting resistance pathways such as PI3K/AKT/mTOR (mammalian target of rapamycin) and CDK4/6, as well as the potential of mTOR and CDK4/6 inhibitors in reshaping the standard of endocrine treatment.^8^ Additionally, they shed light on the crucial role of ATF2 in endocrine resistance by modulating estrogen receptor expression and activity, underscoring the importance of understanding the molecular underpinnings of resistance mechanisms.^21^ Furthermore, the research elucidates the complex signaling mechanisms involved in MMP-3 expression in response to IL-1β, highlighting the significance of ATF-2 and ERK1 in this process.^29^ In the context of extra-terminal motif inhibitors (BETi), one study demonstrates their activation of ATF2 through the JNK1/2 pathway, with ATF2 playing a dual role in attenuating the anti-tumor effects of BETi by modulating ferroptosis and increasing nuclear factor erythroid 2-related factor 2 (NRF2) expression. This finding suggests a novel therapeutic strategy that involves targeting ATF2 or NRF2 in combination with BETi to improve cancer treatment outcomes.^49^ Moreover, the study on microRNAs highlights their role in regulating Ras Suppressor Protein 1 and PINCH1, further underscoring the complexity of the molecular pathways involved in adhesion and survival signaling in breast cancer. Understanding the intricate interplay between MicroRNAs and these key proteins is essential for advancing our knowledge of breast cancer pathophysiology.^50^ Together, these recent findings have the potential to drive innovations in breast cancer treatment and contribute to more effective and targeted therapies.

### Role of ATF2 in endocrine treatment of breast cancer

In a study, it was discovered that pATF2 is a reliable predictor of prognosis and overall survival in patients with ER-positive breast cancer who were subjected to tamoxifen treatment.^20^ Furthermore, when ATF2 was silenced, the growth-inhibitory effects of tamoxifen were reduced in the tamoxifen-sensitive, ER-positive MCF7 cell line. Tamoxifen therapy also caused ATF2 to be phosphorylated in its activation domain in a dose-dependent manner, which increased its transcriptional activity. These results suggested that ATF2 may play a tumor-suppressive role in ER-positive breast cancer.^20^ Similarly, ATF2 has been reported to have both oncogenic and tumor-suppressive properties in skin carcinogenesis, demonstrating its apparent dual role.^51,52^ Therefore, a study was conducted to study the role of ATF2 in resistance to endocrine treatment.^21^ This study used an *in vitro* model of breast cancer and produced interesting findings. It basically tested the knockdown of ATF2 and its effect on two main cell types, MCF7 and TAMR cells. The latter have higher HER-2 protein levels and better ERK1/2 activity than the former. A more important difference is that TAMR cells were tamoxifen resistant, unlike MCF7 cells. Other variants of MCF7 cells that were also tamoxifen-resistant, such as LCC2 and LCC9 cells, were also tested. The results showed that when ATF2 was silenced, the growth of MCF7 parental cells was not affected. However, there was a substantial decrease in the growth of TAMR, LCC2, and LCC9 cells. Additionally, silencing ATF2 inhibited the migration and colony formation of TAMR, LCC2, and LCC9 cells. Knocking down ATF2 increased the levels of estrogen receptor (ER) and ER-regulated genes in TAMR cells compared to MCF7 cells. The R target genes that were mainly affected were TFF1, GREB1, NCOA3, and PGR. Furthermore, differential pathway analysis confirmed that knocking down ATF2 increased ER activity in TAMR cells. Gene expression analysis also revealed that several genes known to cause tamoxifen resistance were differentially regulated by ATF2 in TAMR cells compared to parental MCF7 cells. These results may suggest that ATF2 targets pathways associated with tamoxifen resistance. These findings also suggest that inhibiting ATF2 may be a way to overcome endocrine resistance and demonstrate the dual role of this transcription factor in regulating both endocrine sensitivity and resistance by modulating ER expression and activity.^21^

## Conclusion

In this review, we explored the topic of ATF2 and its role in the development of breast cancer. It is evident that the expression and phosphorylation of ATF2 is necessary for numerous cellular functions, such as cell growth, proliferation, inflammation, death, and DNA damage and repair. The phosphorylation and regulation of ATF2 is also accomplished through various signaling pathways, residues, and protein kinases. There is still uncertainty regarding the exact role of ATF2 in the development and outcome of breast cancer. Several studies suggest that ATF2 may function as an oncogene, worsening the outcome of the disease, as it increases the transcription of genes such as MMP2, MMP13, cyclin A, and aromatase that contribute to the progression and metastasis of breast cancer. Other research has found that the expression of ATF2 plays a role in improving the outcome of breast cancer. It has been identified as a predictor of improved overall survival in patients with certain types of breast cancer in the presence of tamoxifen treatment. Overall, we believe that more research needs to be done on this topic using the resources and claims we mentioned as bases for upcoming advancements. Progress needs to be made to clarify the uncertainty surrounding the exact role of ATF2 and its different behaviors and outcomes in different types of cancers.

In conclusion, our review has revealed several key insights, including that ATF2 may have dual functions in the development of breast cancer. These findings have important implications for the development of therapeutic drugs that target the pathways involved in the progression of the disease. Through our review, we have identified several areas for further research, such as the exact role of ATF2 in endocrine resistance and the pathways that may be susceptible to endocrine treatment. Overall, this review highlights the significance of ATF2 at the molecular level in breast cancer progression and underscores the need for continued investigation in this area. As we have shown, several clinical trials are ongoing in this regard that may yield very promising results for the therapeutic treatment of breast cancer.
